# Supplementary material for: HIVprotI: an integrated web based platform for prediction and design of HIV proteins inhibitors
Source: J Cheminform. 2018 Mar 9;10:12. doi: 10.1186/s13321-018-0266-y (PMC5845081; doi:10.1186/s13321-018-0266-y)
Supplement: Supplementary file 2 — Additional file 2. Source code of HIVProtI web server. [file 13321_2018_266_MOESM2_ESM.zip › HIVprotI_Source-code/hivprotI/weka-3-6-6/doc/weka/filters/unsupervised/attribute/package-summary.html]

weka.filters.unsupervised.attribute


---


|  |  |  |
| --- | --- | --- |
| **Overview** \| **Package** \| Class \| **Tree** \| **Deprecated** \| **Index** \| **Help** \| **Weka's home** | |  |
| **PREV PACKAGE**   **NEXT PACKAGE** | **FRAMES**    **NO FRAMES**     **All Classes** |  |


---

## Package weka.filters.unsupervised.attribute

| **Class Summary** | |
| --- | --- |
| **AbstractTimeSeries** | An abstract instance filter that assumes instances form time-series data and performs some merging of attribute values in the current instance with attribute attribute values of some previous (or future) instance. |
| **Add** | An instance filter that adds a new attribute to the dataset. |
| **AddCluster** | A filter that adds a new nominal attribute representing the cluster assigned to each instance by the specified clustering algorithm. |
| **AddExpression** | An instance filter that creates a new attribute by applying a mathematical expression to existing attributes. |
| **AddID** | An instance filter that adds an ID attribute to the dataset. |
| **AddNoise** | An instance filter that changes a percentage of a given attributes values. |
| **AddValues** | Adds the labels from the given list to an attribute if they are missing. |
| **Center** | Centers all numeric attributes in the given dataset to have zero mean (apart from the class attribute, if set). |
| **ChangeDateFormat** | Changes the date format used by a date attribute. |
| **ClassAssigner** | Filter that can set and unset the class index. |
| **ClusterMembership** | A filter that uses a density-based clusterer to generate cluster membership values; filtered instances are composed of these values plus the class attribute (if set in the input data). |
| **Copy** | An instance filter that copies a range of attributes in the dataset. |
| **Discretize** | An instance filter that discretizes a range of numeric attributes in the dataset into nominal attributes. |
| **FirstOrder** | This instance filter takes a range of N numeric attributes and replaces them with N-1 numeric attributes, the values of which are the difference between consecutive attribute values from the original instance. |
| **InterquartileRange** | A filter for detecting outliers and extreme values based on interquartile ranges. |
| **KernelFilter** | Converts the given set of predictor variables into a kernel matrix. |
| **MakeIndicator** | A filter that creates a new dataset with a boolean attribute replacing a nominal attribute. |
| **MathExpression** | Modify numeric attributes according to a given expression   Valid options are: |
| **MergeTwoValues** | Merges two values of a nominal attribute into one value. |
| **MultiInstanceToPropositional** | Converts the multi-instance dataset into single instance dataset so that the Nominalize, Standardize and other type of filters or transformation can be applied to these data for the further preprocessing.  Note: the first attribute of the converted dataset is a nominal attribute and refers to the bagId. |
| **NominalToBinary** | Converts all nominal attributes into binary numeric attributes. |
| **NominalToString** | Converts a nominal attribute (i.e. |
| **Normalize** | Normalizes all numeric values in the given dataset (apart from the class attribute, if set). |
| **NumericCleaner** | A filter that 'cleanses' the numeric data from values that are too small, too big or very close to a certain value (e.g., 0) and sets these values to a pre-defined default. |
| **NumericToBinary** | Converts all numeric attributes into binary attributes (apart from the class attribute, if set): if the value of the numeric attribute is exactly zero, the value of the new attribute will be zero. |
| **NumericToNominal** | A filter for turning numeric attributes into nominal ones. |
| **NumericTransform** | Transforms numeric attributes using a given transformation method. |
| **Obfuscate** | A simple instance filter that renames the relation, all attribute names and all nominal (and string) attribute values. |
| **PartitionedMultiFilter** | A filter that applies filters on subsets of attributes and assembles the output into a new dataset. |
| **PKIDiscretize** | Discretizes numeric attributes using equal frequency binning, where the number of bins is equal to the square root of the number of non-missing values.    For more information, see:    Ying Yang, Geoffrey I. |
| **PotentialClassIgnorer** | This filter should be extended by other unsupervised attribute filters to allow processing of the class attribute if that's required. |
| **PrincipalComponents** | Performs a principal components analysis and transformation of the data.  Dimensionality reduction is accomplished by choosing enough eigenvectors to account for some percentage of the variance in the original data -- default 0.95 (95%).  Based on code of the attribute selection scheme 'PrincipalComponents' by Mark Hall and Gabi Schmidberger. |
| **PropositionalToMultiInstance** | Converts the propositional instance dataset into multi-instance dataset (with relational attribute). |
| **RandomProjection** | Reduces the dimensionality of the data by projecting it onto a lower dimensional subspace using a random matrix with columns of unit length (i.e. |
| **RandomSubset** | Chooses a random subset of attributes, either an absolute number or a percentage. |
| **RELAGGS** | A propositionalization filter inspired by the RELAGGS algorithm.  It processes all relational attributes that fall into the user defined range (all others are skipped, i.e., not added to the output). |
| **Remove** | A filter that removes a range of attributes from the dataset. |
| **RemoveType** | Removes attributes of a given type. |
| **RemoveUseless** | This filter removes attributes that do not vary at all or that vary too much. |
| **Reorder** | A filter that generates output with a new order of the attributes. |
| **ReplaceMissingValues** | Replaces all missing values for nominal and numeric attributes in a dataset with the modes and means from the training data. |
| **Standardize** | Standardizes all numeric attributes in the given dataset to have zero mean and unit variance (apart from the class attribute, if set). |
| **StringToNominal** | Converts a string attribute (i.e. |
| **StringToWordVector** | Converts String attributes into a set of attributes representing word occurrence (depending on the tokenizer) information from the text contained in the strings. |
| **SwapValues** | Swaps two values of a nominal attribute. |
| **TimeSeriesDelta** | An instance filter that assumes instances form time-series data and replaces attribute values in the current instance with the difference between the current value and the equivalent attribute attribute value of some previous (or future) instance. |
| **TimeSeriesTranslate** | An instance filter that assumes instances form time-series data and replaces attribute values in the current instance with the equivalent attribute values of some previous (or future) instance. |
| **Wavelet** | A filter for wavelet transformation.    For more information see:    Wikipedia (2004). |

---


|  |  |  |
| --- | --- | --- |
| **Overview** \| **Package** \| Class \| **Tree** \| **Deprecated** \| **Index** \| **Help** \| **Weka's home** | |  |
| **PREV PACKAGE**   **NEXT PACKAGE** | **FRAMES**    **NO FRAMES**     **All Classes** |  |


---
